# Supplementary material for: Revisiting inference for ARMA models: Improved fits and superior confidence intervals
Source: PLoS One. 2025 Oct 24;20(10):e0333993. doi: 10.1371/journal.pone.0333993 (PMC12551883; doi:10.1371/journal.pone.0333993)
Supplement: S1 Appendix — An appendix demonstrating why uniform sampling of ARMA model parameters is impractical. (PDF) [file pone.0333993.s001.pdf]

S1 Appendix: Uniform Sampling

A common approach to optimizing a non-convex loss function is to perform the optimization routine with distinct parameter initializations. For ARMA models, picking suitable initialization is a challenging problem that we address with Algorithm 1. An alternative approach would involve sampling each coefficient independently. To see why an independent sampling scheme is not used, consider an AR(2) model. An initialization with parameters $(\phi_1, \phi_2) = (1.1, 0.1)$ is not a valid initialization—as the polynomial roots lie outside the complex unit circle—whereas $(\phi_1, \phi_2) = (1.1, -0.2)$ is perfectly acceptable. Our algorithm accounts for the complex relationship between model parameters when obtaining random initializations.

To visualize why an independent sampling scheme is not used, consider sampling parameters from a Uniform$(-1, 1)$ distribution. In Fig S1, we plot inverted roots that are a result of sampling from this distribution for AR(2) and AR(3) models. The figure illustrates that a significant percentage of uniformly sampled parameter initializations lie outside the accepted region, and, critically, the entire region of possible initializations is not well covered by uniform sampling. Picking a uniform distribution with different bounds—or any other independent sampling distribution—results in similar problems. In order to uniformly sample from the possibly regions, it is necessary to account for the geometry of parameter space, a problem solved by Algorithm 1.

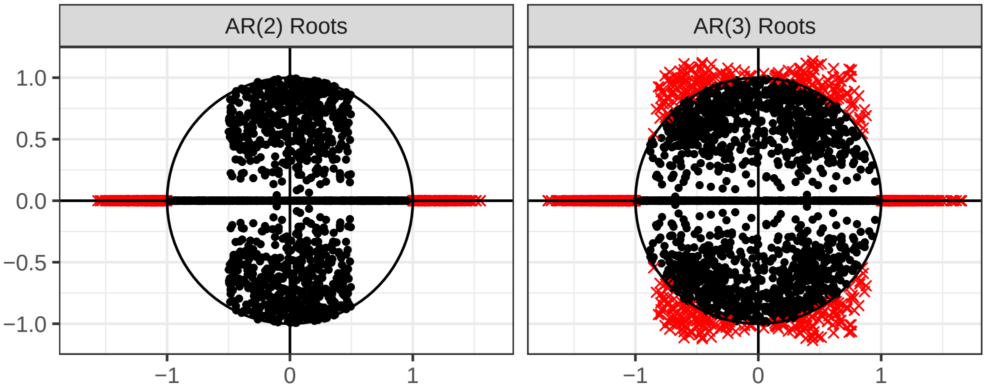

Fig S1: Inverted roots of 1000 samples of AR(2) and AR(3) coefficients sampled independently from a U$(-1, 1)$ distribution. The red "x"s are points that lie outside the accepted region, which represents 12.6% of the AR(2) coefficients and 24.2% of the AR(3) coefficients. Increasing the width of the uniform sampling distribution results in a larger fraction outside the accepted region, and decreasing the width results in worse coverage of the range of acceptable parameter values.
