# Supplementary material for: Revisiting inference for ARMA models: Improved fits and superior confidence intervals
Source: PLoS One. 2025 Oct 24;20(10):e0333993. doi: 10.1371/journal.pone.0333993 (PMC12551883; doi:10.1371/journal.pone.0333993)
Supplement: S2 Appendix — An appendix comparing our algorithm to existing Python software. (PDF) [file pone.0333993.s002.pdf]

S2 Appendix: Python Comparison

Although Algorithm 1 is language agnostic, our implementation is in R. Consequently, all simulation studies for this article use R's `stats::arima` function for baseline comparisons. To demonstrate applicability to other software environments, we briefly compare model likelihoods fit using Python's `statsmodels.tsa` module against our implementation of Algorithm 1. We generate 100 unique Gaussian ARMA$(2, 1)$ models and datasets (each with $n = 100$) where R's `stats::arima` provides sub-optimal estimates. These observations are used to fit ARMA$(2, 1)$ models in Python. Despite these datasets being chosen for sub-optimal results in R, the log-likelihoods in both R and Python are roughly equivalent—a result of both software packages using the same general approach to fitting model parameters.

Our implementation of Algorithm 1 resulted in higher log-likelihoods for 97 out of the 100 datasets compared to Python. The log-likelihood deficiencies for the remaining three datasets were all smaller than $\epsilon = 10^{-5}$, which is smaller than the tolerance level to be considered as an improvement in our other simulation studies. While potentially insignificant, these differences can be eliminated by increasing the number of parameter initializations and the convergence criteria of our algorithm. Directly implementing our algorithm in Python would further eliminate the possibility of these discrepancies entirely.
