# Supplementary material for: Revisiting inference for ARMA models: Improved fits and superior confidence intervals
Source: PLoS One. 2025 Oct 24;20(10):e0333993. doi: 10.1371/journal.pone.0333993 (PMC12551883; doi:10.1371/journal.pone.0333993)
Supplement: Attachment — Submitted filename: ARMA initialization.pdf [file pone.0333993.s004.pdf]

Referee report for "Revisiting inference for ARMA models: Improved fits and superior confidence intervals"

The paper addresses an important yet underappreciated issue: the presence of multiple local maxima in the log-likelihood function of ARMA time series models. Standard estimation algorithms overlook this, potentially resulting in suboptimal parameter estimates by converging to local rather than global maxima.

To mitigate this, the authors propose initializing the optimization algorithm at multiple starting points. The best parameter estimates are then selected based on the highest log-likelihood achieved across all initializations. This approach is logical and well-motivated.

A key challenge in implementing this strategy is that valid AR and MA parameter values must satisfy stationarity and invertibility conditions, which are governed by the roots of associated polynomials. The authors tackle this issue by proposing a method for randomly sampling these roots, thereby generating admissible initial values.

I have two main criticisms of the paper, outlined below, along with suggested solutions that should be feasible to implement with reasonable effort.

1) The roots of the polynomials that one has to consider may be real or complex. Sampling only complex roots can result in only part of the parameter space being covered, as shown by the authors through a simple example. Similar problems arise if the roots are real and sampled with the same sign. To try to address this issue, the authors propose an algorithm (for example, the real root pairs are sampled as real with probability $1/\sqrt{2}$) that is extremely ad-hoc, with no rationale behind it. However, a simple solution already exists for this problem, given by Theorem 2 in "The restricted likelihood ratio test for autoregressive processes" by Chen and Deo, Journal of Time Series Analysis, 2012. This Theorem 2 in Chen and Deo (2012) shows that the vector of partial autocorrelations $\boldsymbol{\phi} = (\phi_{11}, \phi_{22}, ..., \phi_{pp})$ lying in the hypercube $(-1, 1] \times (-1, 1)^{p-1}$ corresponds to the set of all AR(p) coefficients $\boldsymbol{\alpha} = (\alpha_1, \alpha_2, ..., \alpha_p)$ that are stationary or have exactly one unit root. Note that the unit root is approached only as ϕ_{11}

approaches 1. Furthermore, equations (4) and (5) in Chen and Deo (2012) show how to convert any vector $\boldsymbol{\phi}$ to its corresponding $\boldsymbol{\alpha}$ and vice versa. Thus, a straightforward way to solve the initialization problem is to randomly sample ϕ_{11} from $U(-1+\gamma, 1-\gamma)$ and, for $i = 2, ..., p$, sample each ϕ_{ii} independently from $U(-1+\theta, 1-\theta)$, where $0 < \gamma < \theta$ are sufficiently small, and then convert the resulting vector $\boldsymbol{\phi}$ to the corresponding vector $\boldsymbol{\alpha}$. (Indeed, the optimization could be done entirely in the $\boldsymbol{\phi}$ space without having to transform to the $\boldsymbol{\alpha}$ space).

The same re-parameterization above also holds in an analogous manner for the MA coefficients.

2) In practically any real life application of ARMA models, the mean of the series is not known but is non-zero. This is obviously the case for the "annual depths of Lake Michigan" data analysed in the paper, as seen in Figure 5. The standard practice is to subtract the sample mean from the data and then treat the resulting mean-adjusted data as having a zero mean. In a series of papers, Chen and Deo (Econometric Theory 2009, Biometrika 2010, Journal of Time Series Analysis 2012) showed that this has a non-trivial negative impact on the AR parameter estimators near the unit root, which remains even asymptotically. In these series of papers, Chen and Deo showed that the restricted likelihood (REML) estimators of the process do not suffer from this degradation, following on the work of Cheang and Reinsel (2000). Consequently, the authors of the paper under review should demonstrate their findings for the REML likelihood for ARMA models; as shown in "Analysis of ecological time series with ARMA(p,q) models" by Ives, Abbott and Ziebarth (2010), even the REML log likelihood suffers from multi-model behaviour. Hence, the authors of the current study would provide a service to the research community by providing a feasible algorithm that uses the method suggested in (1) above, in conjunction with the REML likelihood, to estimate ARMA models.
