# Supplementary material for: Revisiting inference for ARMA models: Improved fits and superior confidence intervals
Source: PLoS One. 2025 Oct 24;20(10):e0333993. doi: 10.1371/journal.pone.0333993 (PMC12551883; doi:10.1371/journal.pone.0333993)
Supplement: Attachment — Submitted filename: PONE-D-25-22432_comments_LU.pdf [file pone.0333993.s005.pdf]

Review for PONE-D-25-22432 "Revisiting Inference for ARMA Models: Improved Fits and Superior Confidence Intervals" by Jesse Wheeler and Edward L. Ionides

This manuscript makes a significant contribution to time series analysis by addressing a critical flaw in ARMA model estimation, proposing a novel multi-start algorithm to improve parameter estimation, and demonstrating the superiority of profile likelihood confidence intervals over those based on Fisher's information matrix. The simulation studies and real-world example provide robust evidence of the method's efficacy, and the work is implemented in the R package `arima2`, enhancing its practical utility. Overall, the paper is well-written, and the topic aligns well with the journal's scope. However, the manuscript requires several clarifications. I therefore recommend acceptance once the comments below are appropriately addressed.

1. In Section 2.2, on Page 6, lines 196–198, the authors mention that 36,000 unique time series were generated. Does this mean that 1,000 independent time series were generated for each combination of n, p, and q? Were the model parameters fixed or randomly generated? A brief clarification in the manuscript would be helpful.

2. In Figure 1, the authors demonstrate optimization issues—i.e., sub-optimal parameter estimates—in four generated MA(1) models. However, AR(p) models for $p = 1, 2, 3$ and MA(q) models for $q = 1, 2, 3$ are not included in the simulation results shown in Figure 2. Could the authors consider adding these models to Figure 2 to present a more complete set of ARMA(p, q) models with $p, q \leq 3$?

3. In Section 2.2, on Page 7, lines 228–229, the authors mention that the average computation time for obtaining the MLEs was 0.6 seconds using the proposed method. What is the corresponding average computation time using the standard approach for ARMA parameter estimation? Including a table or figure comparing the runtimes of `stats::arima` versus `arima2::arima` across different sample sizes would enhance the practical relevance of the discussion. Additionally, providing the hardware specifications (e.g., processor, RAM) would help clarify the computational context.

4. In Figure 3A, were the confidence intervals based on Fisher's information matrix constructed using the standard approach or the proposed method? Additionally, does the `arima2` package provide a function for computing profile likelihood confidence intervals (PLCIs)?

5. In Table 2, were the ARMA(2,1) parameters estimated using the standard approach or the proposed method? Does `arima2::arima` include an invertibility check?

6. The caption for Table 1 should be moved above the table to be consistent with Table 2.

7. On Page 3, line 92, “do to” should be corrected to “due to”

8. For the annual depths of Lake Michigan example, could the authors include the ACF and PACF plots of the time series, as well as the ACF plot of the residuals, to aid in determining the appropriate ARMA orders p and q?
