# Supplementary material for: Revisiting inference for ARMA models: Improved fits and superior confidence intervals
Source: PLoS One. 2025 Oct 24;20(10):e0333993. doi: 10.1371/journal.pone.0333993 (PMC12551883; doi:10.1371/journal.pone.0333993)
Supplement: Attachment — Submitted filename: response-to-reviewers.pdf [file pone.0333993.s006.pdf]

Journal Requirements

1. Please ensure that your manuscript meets PLOS ONE's style requirements, including those for file naming.

We apologize for not following these instructions in the original submission. To meet this requirements, the following changes were made:

- Added section "Material and Methods" (Section 2) and "Results" (Section 3). The previous section "2 Maximum Likelihood for ARMA models" was nested as subsection 2.1 in "Material and Methods"; subsection "2.2 Simulation Studies" was moved to subsection "3.1 Simulation Studies"; and finally Section "3 Annual Depths of Lake Michigan" was moved to "3.2 Annual Depths of Lake Michigan". For reference, a detailed structure of the new Section headings is provided as a response to Reviewer 1.
- Files have been renamed to follow submission requirements.

We are unable to open your Supporting Information files [sourceFiles.zip, arima2_3.3.0.tar.gz] Please kindly revise as necessary and re-upload.

The original supplement file contained both scripts and `.rds` data files within the same zipped folder. For clarity, we have instead uploaded two distinct `.zip` folders, one containing the simulation study scripts, the other containing the results of these scripts. The primary data structure is R data files (`.rds` files), however there are also two `.csv` files that are used with Python related scripts.

The supporting information file `arima2_3.4.0.tar.gz` (which is the updated version of the object `arima2_3.3.0.tar.gz`) contains the compiled source code for the `arima2` R package; it is not designed or intended to be opened, rather it contains R package source code that can be installed by R users. We provided this compiled source code as a courtesy so that interested readers may have easy access to the exact version of the package that was used in the article, though if it is inconvenient for the journal submission format, we can remove these files. Users may still download the most recent version of the package CRAN using `install.packages('arima2')`, and the same version used in the manuscript by searching CRAN archives.

Reviewer 1

The manuscript is clear and sound all the graphs and results are properly explained although the format of the paper is unusual you should specicify the literature review section also follow the proper layout of a research paper.Section 1 introduction section 2 literature review 3 methodoogy 4 results and discussions 5 Conclusion then followed by your appendix

Thank you for this thoughtful summary and suggestion. In order to comply with journal requirements, we have edited the article so that the article structure is as follows:

1 Introduction

2 Material and Methods

- 2.1 Maximum Likelihood for ARMA Models
- 2.2 Novel Multi-start Algorithms

3 Results

- 3.1 Simulation Studies
  - 3.1.1 Parameter Uncertainty
  - 3.1.2 AIC Table Consistency
- 3.2 Annual Depths of Lake Michigan
  - 3.2.1 Parameter Uncertainty

4 Discussion

This new section structure follows the journal requirements. As for a literature review, PLOS ONE does not strictly require a section dedicated to this topic. Our article follows the standard practice in statistics articles, which is to include the literature review as part of the introduction, as well including citations to existing literature when applicable throughout the remainder of the article.

Referee 2

Summary: *The paper is very nice and tries to address an important and not adequately recognized problem. However, the solution provided by the authors is ad-hoc. That said, there is an available result in the literature that allows the authors to address this criticism and a revised paper will be suitable for publication.*

Thank you for the positive feedback and recommendations. The paper has been revised to address the reviewer's concerns. Below we include a detailed response to the itemized concerns that were included in an attached document.

Detailed Comments: *The paper addresses an important yet underappreciated issue: the presence of multiple local maxima in the log-likelihood function of ARMA time series models. Standard estimation algorithms overlook this, potentially resulting in suboptimal parameter estimates by converging to local rather than global maxima.*

To mitigate this, the authors propose initializing the optimization algorithm at multiple starting points. The best parameter estimates are then selected based on the highest log likelihood achieved across all initializations. This approach is logical and well-motivated.

A key challenge in implementing this strategy is that valid AR and MA parameter values must satisfy stationarity and invertibility conditions, which are governed by the roots of associated polynomials. The authors tackle this issue by proposing a method for randomly sampling these roots, thereby generating admissible initial values.

I have two main criticisms of the paper, outlined below, along with suggested solutions that should be feasible to implement with reasonable effort.

(1) The roots of the polynomials that one has to consider may be real or complex. Sampling only complex roots can result in only part of the parameter space being covered, as shown by the authors through a simple example. Similar problems arise if the roots are real and sampled with the same sign. To try to address this issue, the authors propose an algorithm (for example, the real root pairs are sampled as real with probability $p = \sqrt{1/2}$) that is extremely ad-hoc, with no rationale behind it.

However, a simple solution already exists for this problem, given by Theorem 2 in "The restricted likelihood ratio test for autoregressive processes" by Chen and Deo, Journal of Time Series Analysis, 2012. This Theorem 2 in Chen and Deo (2012) shows that the vector of partial autocorrelations $\boldsymbol{\phi} = (\phi_{11}, \phi_{22}, \ldots, \phi_{pp})$ lying in the hypercube $(-1, 1] \times (-1, 1)^{p-1}$ corresponds to the set of all AR(p) coefficients $\boldsymbol{\alpha} = (\alpha_1, \alpha_2, \ldots, \alpha_p)$ that are stationary or have exactly one unit root. Note that the unit root is approached only as ϕ_{11} approaches 1. Furthermore, equations (4) and (5) in Chen and Deo (2012) show how to convert any vector $\boldsymbol{\phi}$ to its corresponding α and vice versa. Thus, a straightforward way to solve the initialization problem is to randomly sample ϕ_{11} from $U(-1+\gamma, 1-\gamma)$ and, for $i = 2, \ldots, p$, sample each ϕ_{ii} independently from $U(-1+\theta, 1-\theta)$, where $0 < \gamma < \theta$ are sufficiently small, and then convert the resulting vector $\boldsymbol{\phi}$ to the corresponding vector $\boldsymbol{\alpha}$. (Indeed, the optimization could be done entirely in the ϕ space without having to transform to the $\boldsymbol{\alpha}$ space). The same re-parameterization above also holds in an analogous manner for the MA coefficients.

Thank you for this insightful comment. We are aware of the equations (4) and (5) in [1], which are often referred to as the Durbin-Levinson recursions. These transformations already play a major role in our software package. Similar to what you mentioned, if the default argument `transform.pars = TRUE` to the `arima2::arima` function is used, then the optimization occurs in the ϕ space.

Per your recommendation, we have added a Durbin-Levinson initialization scheme to our software package that can be used to randomly sample ARMA model parameters within the admissible region. This option can be used by setting `init_method = "DL"` in the `arima2::arima` function, or by setting `method = "DL"` in the `arima2::sample_ARMA_coef` function. We have also added Algorithm 2 to the manuscript, which is analogous to Algorithm 1 but using the suggested Durbin-Levinson recursion for initializing parameters rather than the uniform-roots sampling scheme. However, we have elected to retain the original root-sampling algorithm in both the software and manuscript for the following reasons.

Our extensive testing indicates that both algorithms yield similar improvements over the single initialization approach. However, the originally proposed uniform-root sampling scheme (Algorithm 1) leads to slightly higher rates of improvement in the log-likelihood compared the the Durbin-Levinson sampling scheme (Algorithm 2). Notably, this advantage persists even when the simulated data are generated from models whose parameters are randomly sampled using the Durbin-Levinson algorithm, a scenario that would be expected to favor Algorithm 2. Specifically, after re-running the experiment using 36,000 unique time-series models and data sets, Algorithm 1 improved upon the baseline in 20.72% of simulations (this is a slight change from the reported 20.8% in the original manuscript due to re-conducting the experiment with a new reproducible seed and sampling scheme), whereas Algorithm 2 improved upon the baseline in 20.35% of the simulated data. In a direct comparison—using a log-likelihood difference of 0.001 as the threshold for measuring improvements—Algorithm 1 resulted in larger log-likelihoods than Algorithm 2 in 5.31% of all simulated data, whereas Algorithm 2 resulted in a larger log-likelihood in 4.88% of the simulations. We have no personal preference for using Algorithm 1 or Algorithm 2, other than our stated goal which is to improve maximum likelihood estimation. Our software now includes the ability use both methods, but our experiments suggest that Algorithm 1 has a slight advantage towards achieving our goal, so it remains the primary focus of our manuscript.

Additionally, we do not agree with the characterization of Algorithm 1 as "extremely ad-hoc, with no rationale behind it". The set of admissible parameter values is governed by the behavior of the corresponding polynomial roots, a fact that is not in question. Because the inverted roots of all valid initializations must lie in the complex unit-circle, to us it seems that uniformly sampling from the root space is a rational and simple approach. Trivially, the transformation from root-space to the target parameter space is both one-to-one and onto, as this is precisely how the target space is defined. Of course, as we note, uniformly sampling from the root space leads to poor representation in the parameter space because some parameters will always have the same sign unless root pairs are real. Thus, the choice of $p = \sqrt{1/2}$ implies that the product of root pairs is positive with probability 1/2, and because model parameters are determined by the sum of products of root pairs, this results in equal chance that the leading AR/MA coefficients are positive or negative. Interestingly, we found that this sampling scheme results in real roots at roughly the same rate as when parameters are initialized using the Durbin-Levinson recursions.

Though Theorem 2 of [1] shows correspondence between the space of partial autocorrelations and the target parameter space, uniform sampling of the partial autocorrelations does not imply uniform coverage in the target space (the same is true when uniformly sampling in the root space). Ultimately, whether parameters are initialized by sampling from the root space or from the partial autocorrelation space is unimportant, as long as the resulting set of parameters is within the admissible region in order to try multiple parameter initializations to combat the multi-modality of the likelihood surface. We are not aware of any theoretical reason to believe that uniform sampling for one parameterization is preferable to any other type of sampling for any other transformation of the parameter space.

> *(2) In practically any real life application of ARMA models, the mean of the series is not known but is non-zero. This is obviously the case for the "annual depths of Lake Michigan" data analysed in the paper, as seen in Figure 5. The standard practice is to subtract the sample mean from the data and then treat the resulting mean-adjusted data as having a zero mean.*

While the approach you mention is similar, it is distinct from the method implemented in our package and in the `stats::arima` function that our package is built from. In both cases, the process is assumed to have a non-zero mean—denoted here as β_0—which is estimated, and is not equal to the sample mean as claimed. Instead, β_0 is estimated as a regression coefficient via maximum likelihood. For technical details, we refer you to the internal `armafn` function, which is built within the `stats::arima` and `arima2::arima` functions. This approach bypasses the need to subtract the data mean and instead derives an optimal constant directly from the likelihood function. We estimate that this approach is the most common among practitioners who use ARMA models, as this same algorithm is also standard in Python implementations (e.g., in the commonly used `statsmodels` package). With the above caveat noted, we still acknowledge that the same issues you mention below may be present when estimating the mean as described, and that REML estimators may still have some practical advantages over maximum likelihood estimators.

> *In a series of papers, Chen and Deo (Econometric Theory 2009, Biometrika 2010, Journal of Time Series Analysis 2012) showed that this has a non-trivial negative impact on the AR parameter estimators near the unit root, which remains even asymptotically. In these series of papers, Chen and Deo showed that the restricted likelihood (REML) estimators of the process do not suffer from this degradation, following on the work of Cheang and Reinsel (2000). Consequently, the authors of the paper under review should demonstrate their findings for the REML likelihood for ARMA models; as shown in "Analysis of ecological time series with ARMA*(p, q) *models" by Ives, Abbott and Ziebarth (2010), even the REML log likelihood suffers from multi-modal behaviour. Hence, the authors of the current study would provide a service to the research community by providing a feasible algorithm that uses the method suggested in (1) above, in conjunction with the REML likelihood, to estimate ARMA models.*

Thank you for the references and suggestions. REML estimators for ARMA models is a relevant and practical topic of which we were previously unaware. We have revised the manuscript to include some of the suggested references, resulting in an improved manuscript. References to REML estimators have been added to Section 2.1:

> *Given the importance of ARMA models, numerous methods have been developed for parameter estimation. For instance, parameters can be estimated using Bayesian inference techniques [2, 3], neural networks [4], or by maximizing restricted likelihood [5, 6, 1], among other approaches. Specialized methods include those for integer-valued data [7], approaches robust to outliers [8], and non-Gaussian ARMA processes [9]. While each methodology has its merits, we focus on the approach most widely adopted in statistical practice: likelihood maximization, under the assumption that the WN process* $\{w_t\}$ *is Gaussian.*

We have also included REML estimators as part of the Discussion (Section 4):

> *Parameter estimates corresponding to higher likelihood values are not necessarily scientifically preferable to alternative regions of parameter space with lower likelihood values [10]. Sometimes, our improved estimates may result in models with nearly canceling roots, parameters near boundary conditions, or otherwise unfavorable statistical properties. On other occasions, our method can rescue a naive optimization attempt from a local maximum having those unfavorable properties. Alternative forms of parameter estimation may also be preferable in some cases. For example, a series of papers by Chen and Deo show that restricted likelihood estimators have favorable properties compared to maximum likelihood estimators when the AR parameters are near the unit root [5, 6, 1]. Practitioners should carefully evaluate fitted models to ensure they are appropriate for the data and problem at hand.*

After reviewing the referenced papers, we agree that addressing the multimodality of REML estimators would be a valuable contribution to the scientific community. However, the inclusion of REML estimators is beyond the scope of the present article, though we are eager to explore this issue in the future.

While REML may offer certain practical advantages over maximum likelihood estimation, maximum likelihood remains the prevailing standard in both scientific research and industry practice. This is reflected in the major statistical software packages available to practitioners. As previously noted, the base statistics packages in both R and Python provide tools for the maximum likelihood estimation of ARIMA model coefficients. Additionally, other widely used data analysis platforms—including SAS, MATLAB, and Excel—also default to maximum likelihood estimation for ARMA models. In our article, we do not discuss these other implementations as precise implementation details are unavailable due to the proprietary nature of these software packages.

As a result of the wide-spread nature of maximum likelihood estimation for ARMA models, our paper addressing the issue of multi-modality in the likelihood surface is a significant contribution to the scientific community. In the Discussion section, we previously mentioned that maximum likelihood estimates are not necessarily the best approach for any given data set and ARMA model, even if it is currently the most common strategy. This discussion has now been expanded to include the provided references on REML estimators and their potential advantages.

Parameter estimates corresponding to higher likelihood values are not necessarily scientifically preferable to alternative regions of parameter space with lower likelihood values [10]. Sometimes, our improved estimates may result in models with nearly canceling roots, parameters near boundary conditions, or otherwise unfavorable statistical properties. On other occasions, our method can rescue a naive optimization attempt from a local maximum having those unfavorable properties. Alternative forms of parameter estimation may also be preferable in some cases. For example, a series of papers by Chen and Deo show that restricted likelihood estimators have favorable properties compared to maximum likelihood estimators when the AR parameters are near the unit root [5, 6, 1]. Practitioners should carefully evaluate fitted models to ensure they are appropriate for the data and problem at hand.

Referee 3

This manuscript makes a significant contribution to time series analysis by addressing a critical flaw in ARMA model estimation, proposing a novel multistart algorithm to improve parameter estimation, and demonstrating the superiority of profile likelihood confidence intervals over those based on Fisher's information matrix. The simulation studies and real-world example provide robust evidence of the method's efficacy, and the work is implemented in the R package arima2, enhancing its practical utility. Overall, the paper is well-written, and the topic aligns well with the journal's scope. However, the manuscript requires several clarifications. I therefore recommend acceptance once the comments below are appropriately addressed.

1. In Section 2.2, on Page 6, lines 196–198, the authors mention that 36,000 unique time series were generated. Does this mean that 1,000 independent time series were generated for each combination of n, p, and q? Were the model parameters fixed or randomly generated? A brief clarification in the manuscript would be helpful.

That is correct, 1000 independent time series were generated for each combination of n, p, and q. These come from 1000 randomly generated sets of ARMA model parameters. The statement on 36,000 unique time series was modified to be the following:

For each combination of (p, q, n), 1000 unique parameter sets are generated, as well as a single time series from each set of parameters. In total, this results in 36,000 unique models and datasets.

2. In Figure 1, the authors demonstrate optimization issues—i.e., sub-optimal parameter estimates—in four generated MA(1) models. However, AR(p) models for p = 1, 2, 3 and MA(q) models for q = 1, 2, 3 are not included in the simulation results shown in Figure 2. Could the authors consider adding these models to Figure 2 to present a more complete set of ARMA(p, q) models with p, q ≤ 3?

Thank you for the recommendation. In our preliminary studies, we originally considered pure MA and AR processes as well, but we ultimately decided not to include them in our larger simulations for a few reasons. The first is that the inclusion of pure AR and MA processes resulted in an over-crowded visualization, distracting from the more interesting results with the mixed models. Additionally, the total number of simulations in our study is already very large, and we were trying to focus our computing efforts on the more interesting cases.

Our preliminary results confirmed our suspicion that multiple parameter initializations is not as important for pure AR and MA processes. Not only do they naturally have fewer parameters, but the likelihood surface of pure AR / MA processes is generally much better behaved. This fact has been noted elsewhere, and is one reason existing literature often focuses on these more simple processes. In our preliminary results, we found that for pure AR processes, less than 1% of simulations required multiple restarts to confidently maximize likelihoods, and roughly 1% of the pure MA processes were improved with multiple restarts. For your benefit, Figure L-1 breaks down these preliminary results further by combination of (n, p, q).

As noted in the article, our extensive simulation study considers 36,000 unique generative models. In order to test confidence intervals (Figure 3 in the manuscript), this requires generating 100

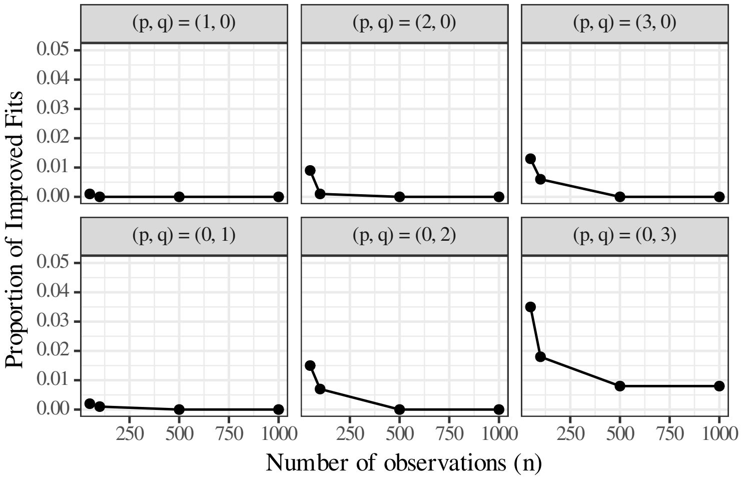

Figure L-1: Preliminary results of pure AR and MA processes.

unique time series from each generative model, resulting in 3,600,000 unique timeseries. For each time-series, we fit the model twice (once using the baseline, once using our approach). This is done for each parameter in order to calculate the profile-confidence interval coverage—one time fixing a parameter, the other time estimating it. Assuming the average of 5 parameters per model (including intercept), this implies fitting more than 36 million models. In reality, because our algorithm requires fitting at a minimum 10 models and returning the best output, the true number of fitted models is more than 360 million.

Even though the algorithm is relatively fast, this large number of fitted models requires thousands of computing hours. Because of this, we felt it was prudent to not include pure AR and MA processes as part of the simulation study as the number of instances that can be improved with multiple initializations appears to be small. We have added the following paragraph in the manuscript to clarify this issue.

> *Likelihood optimization for mixed ARMA models is known to be more challenging than for pure AR or MA processes. Nonetheless, Figure 1 demonstrates that multimodal likelihood surfaces and suboptimal estimates can arise in pure MA(1) processes, and even these simple models can benefit from multiple initializations. This class of models is particularly useful for illustrating the potential for suboptimal estimates, as the presence of only a single parameter allows for easy visualization. Our preliminary results indicate, however, that pure AR and MA processes rarely require multiple initializations for effective optimization. The difficulty of parameter optimization in mixed ARMA models is one reason authors have previously advised caution in their use [11]. Despite the need for caution, mixed models remain commonly used in practice and are therefore the primary focus of our article.*

3. In Section 2.2, on Page 7, lines 228–229, the authors mention that the average computation time for obtaining the MLEs was 0.6 seconds using the proposed method. What is the corresponding average computation time using the standard approach for ARMA parameter estimation? Including a table or figure comparing the runtimes of stats::arima versus arima2::arima across different sample sizes would enhance the practical relevance of the discussion. Additionally, providing the hardware specifications (e.g., processor, RAM) would help clarify the computational context.

Thank you for the recommendation. Summarizing the timing of the algorithms considered in this article can be difficult due to high levels of variability. Not only does it depend on the choice of p, q, and n, each individual time series will have different computing times. Even after fixing the input data, each random initialization will take a distinct amount of time to converge to a local solution. Random initializations closer to a local solution will converge quickly relative to those that are further away. It is for this reason we opted originally to only state average computing time for our new algorithm; because it typically finishes in less than a second, the computing difference between the two algorithms seems practically unimportant. In our revised manuscript, we have included Table 1 that summarizes the mean and standard deviation of computing times, measured in seconds.

4. In Figure 3A, were the confidence intervals based on Fisher's information matrix constructed using the standard approach or the proposed method? Additionally, does the arima2 package provide a function for computing profile likelihood confidence intervals (PLCIs)?

The Fisher's information was constructed using the proposed method. As noted in section 3.1.1, there was no notable difference between the performance of Fisher's confidence intervals when using our proposed algorithm or the accepted standard. To clarify this point, we have amended the caption to read:

Proportion of models that achieved nominal coverage of Bonferroni adjusted 95% confidence intervals. The dashed line denotes the target coverage level. Parameter estimates were obtained using Algorithm 1. (A) Confidence intervals created using Fisher's information matrix. (B) Confidence intervals created using profile likelihoods.

5. In Table 2 (now Table 3) were the ARMA(2,1) parameters estimated using the standard approach or the proposed method? Does arima2::arima include an invertibility check?

The results in Table 2 subtly imply that the parameters reported in Table 3 come from both the standard approach and the proposed method. The caption for Table 3 has been added to clarify this point.

Parameter values of ARMA(p, q) model fit to Lake Michigan-Huron depth data. The same parameters are returned with a single initialization, or when using Algorithms 1 or 2.

Yes, `arima2::arima` does include a final invertibility check. Like the `stats::arima` source code that our package is built around, if the final parameter estimates correspond to a non-invertible ARMA process, then the code applies a transformation before returning the final estimates that guarantees invertibility.

6. The caption for Table 1 (Now Table 2) should be moved above the table to be consistent with Table 2 (Now Table 3)

Thank you for the recommendation, this has been fixed.

7. On Page 3, line 92, "do to" should be corrected to "due to" .

This has been fixed.

8. For the annual depths of Lake Michigan example, could the authors include the ACF and PACF plots of the time series, as well as the ACF plot of the residuals, to aid in determining the appropriate ARMA orders p and q?

Thank you for the recommendation. We have added a new set of figures (Figure 8) that show the ACF and residual diagnostics for the two primary candidate models (ARMA(2, 1) and AR(1)). In doing so, we have created the same set of diagnostic figures shown in Chapter 3.7, "Building ARIMA Models" of Shumway and Stoffer [12]. We have opted not to include more figures, such as PACF and ACF of the original time series and residual diagnostics for all candidate models because we feel that adding more figures will detract from the aim of the article, which is to ensure proper maximization of model likelihood for any fixed choice of p and q, rather selecting p and q.

Referee 4

The paper "Revisiting Inference for ARMA Models: Improved Fits and Superior Confidence Intervals" likely deals with improvements to the estimation and inference procedures for ARMA (AutoRegressive Moving Average) models, focusing on better model fitting and more accurate/confident interval estimation. Here are some question should be answered before publication.

1. How do the new inference techniques affect the stationarity or invertibility conditions of ARMA models?

The new techniques do not have any impact on the stationarity or invertibility conditions of the model. As noted in Section 2.2, we use existing optimization strategies for fitting model parameters, which includes checks for stationarity and invertibility that are part of the `stats::arima` function.

2. How is the accuracy of confidence intervals evaluated?

This is described in detail in Section 3.1.1

For each of the 36,000 generative models from the previous study, we generate 100 additional datasets, estimating the MLE and Bonferroni-adjusted 95% confidence intervals using both estimated standard errors and profile likelihood confidence intervals (PLCIs) from Wilks' theorem. Fig 3 shows ...

The statistics that represent the performance of the confidence intervals are only provided in Figure 3. Here, the caption reads:

Fig 3. Proportion of models that achieved nominal coverage of Bonferroni adjusted 95% confidence intervals. The dashed line denotes the target coverage level. (A) Confidence intervals created using Fisher's information matrix. (B) Confidence intervals created using profile likelihoods.

Using 95% confidence intervals, the nominal coverage should be approximately 95%, so comparing the actual coverage to this target value is a meaningful way to test the accuracy of confidence intervals. To clarify this point, the following sentence has been added to the aforementioned paragraph.

The accuracy of confidence intervals is evaluated by comparing the nominal coverage of the confidence intervals from the simulations to the target 95% coverage level.

3. Can the proposed methods be extended to seasonal or multivariate ARMA models (e.g., SARIMA, VARMA)?

Yes. The second paragraph of the introduction addresses the possibility of our algorithm being used in ARMA extensions:

A commonly used extension of the ARMA model is the integrated *ARMA model, which extends the class of ARMA models to include first or higher order differences. That is, an autoregressive integrated moving average (ARIMA) model is an ARMA model fit after differencing the data in order to make the data stationary. Additional extensions include the modeling of seasonal components (SARIMA), or the inclusion of external regressors (SARIMAX). Our methodology can readily be extended to these model classes as well, but here we focus on ARMA modeling for simplicity.*

4. How does this paper's approach compare to bootstrap or Bayesian interval estimation for ARMA models?

This question is not directly relevant to the article. Our goal is to demonstrate that existing procedures for maximum likelihood estimation often fail to maximize the likelihood, and provide a new procedure that fixes this issue. Neither bootstrap or Bayesian estimates maximize the likelihood function, and therefore are not related to this goal.

While it may be of interest to compare the predictive ability of ARMA models that were fit via maximum likelihood, Bayesian inferences, or otherwise, this comparison is outside the scope of the current manuscript. The goals, advantages, and weaknesses of frequentist vs Bayesian inference are fundamentally different and go beyond simple comparison of their predictive abilities.

5. Does the paper explore asymptotic properties of the new estimators or intervals (e.g., consistency, normality)?

Theoretic properties of the MLE have been well studied and established elsewhere. Our procedure does not come with novel theoretical work regarding its asymptotic properties because it essentially addresses shortcomings in current procedures rather than creating a new type of estimate. Consequently, the existing theory, typically thought to apply to conventional maximum likelihood estimates, is actually more relevant to our estimates. This point is made in several parts of the article, including the discussion, copied below.

A significant motivation for our work is the observation that commonly used statistical software that purports to maximize ARMA model likelihoods fails to do so for a large number of examples. In addition to improving parameter estimates, proper maximization of ARMA model likelihoods is crucial because ARMA models are often used to model serial correlations in regression analyses. In this context, researchers may perform likelihood ratio hypothesis tests for regression coefficients, and the validity of these tests depends on proper likelihood optimization.

This observation also is relevant to model selection, where AIC is often used to select the order of the model.

Since AIC assumes the parameters correspond to maximized likelihoods, enhancements in likelihood maximization can lead to different model selections.

In this context, any theoretical properties that a user would like to rely on when using AIC as a model selection criteria is dependent on the ability to properly maximize the model likelihood, which is the goal of our new estimation procedure.

6. Are there improvements in model diagnostics or residual analysis with the new method?

The process of performing model diagnostics and residual analysis are not impacted by our estima-

tion procedure. However, the results may be more reliable if users decide to use diagnostic tools that assume that the likelihood has been maximized.

7. Does the method improve convergence in the likelihood optimization process? If yes, how?

It is not clear what is meant by "improve convergence". The goal of the algorithm is to increase the probability that the algorithm converges to the global maximum, and in this sense it does improve convergence to the global maximum. As pointed out in the discussion, however, the proposed method also has longer compute times, as it requires repeating a numeric optimization using many starting points. For each starting point, the rate of convergence to a local solution is unchanged, though the repeated search using many starting points does result in longer compute times.

8. What improvements are observed in the width, coverage, or accuracy of these intervals compared to traditional asymptotic intervals?

The answer to this question is summarized in Fig 3. Here, we see the width, coverage, and accuracy of the profile likelihood confidence intervals are all improved compared to the traditional asymptotic intervals.

9. How does the paper ensure that improved fits don't result in overfitting or spurious models?

The improved fits are not directly related to overfitting or spurious models. Our algorithm improves estimates for any fixed model size, so it does not have the potential to result in any overfitting relative to the existing standard. When it comes to selecting the order (p, q) of the ARMA model, overfitting is generally addressed by using AIC and considering other diagnostic plots, as demonstrated in Section 3.2. Our paper does not make any recommended changes to these existing ideas other than improving the estimates for a fixed model size.

10. Are the likelihood surfaces smoother or better-behaved under the new method? Is there any analysis of local minima or optimization difficulty?

The likelihood surface is unchanged. Our approach is to ensure that, given a fixed likelihood surface, the algorithm and software has a high probability to converge to the global maximum rather than local solutions.

11. What is the purpose of initializing parameters ψ, θ using the CSS (Conditional Sum of Squares) estimate? Why not start from random values?

This is discussed in detail in Sections 2.1 and 2.2. In brief, the CSS initialization is fast to compute and often results in parameter initializations that are close to the global maximum. Because of this, it has been the standard approach for more than two decades. However, we demonstrate that using this as the only initialization is not sufficient. Unfortunately, randomly sampling parameter values is a non-trivial task. The problem we directly address with Algorithms 1 and 2 is the ability to randomly sample parameter values. Our approach then is to use a combined strategy: start with the CSS initialization because it is generally quite good, but also try random parameter values using our newly proposed algorithm.

12. How does the probability parameter p (used to sample real vs. complex root pairs) affect the exploration of the parameter space?

We address this question as well in response to comment 1 from reviewer 2. To summarize, the

set of all possible ARMA parameter initializations is defined by the roots of the AR and MA polynomials, defined in section 2.2. The inverse of the roots of these polynomials must lie within the complex unit circle. Thus, we uniformly sample from this space by sampling an angle π and radius r. To ensure that all parameters of the model are real, the roots must come in complex conjugate pairs, so we generally sample two roots at a time. This has the effect, however, that some of the coefficients will always be positive (or negative), because coefficients are formed from the sum of products of root pairs. Therefore, our goal is to ensure that the product of root pairs is positive with probability 1/2, which is achieved by sampling a root pair as real with probability $p = \sqrt{1/2}$. We treat this value as fixed (not a tuning parameter).

> *13. What role does the bound $\gamma \in (0, 0.5)$ play in root sampling, and how does it ensure stationarity and invertibility?*

The inverted roots must be less than 1 in magnitude for stationarity and invertibility. However, behavior of ARMA processes when the root is near unit magnitude becomes problematic. Thus, $\gamma \in (0, 0.5)$ is used to ensure that initializations are not too close to the boundary. For default, we use $\gamma = 0.05$. Similar cutoffs have been used elsewhere, such as the popular `forecast` package in R. The choice of γ has very little affect on the final results.

> *14. In case of an odd number of AR or MA roots, why is the non-paired root sampled only at angle 0 or π?*

This ensures a real root; if the angle is not 0 or π, then the root has a non-zero imaginary part, and the final parameter initialization will not be real.

> *15. What is the significance of sampling root magnitudes from $U(\gamma, 1 - \gamma)$? What would happen if γ were too close to 0 or 0.5?*

See response to question 13.

> *16. How sensitive are the results to the choice of parameters α, γ, and p? Should these be tuned or fixed?*

p should be fixed, and we have modified the algorithm to make this point clear. α and γ do not need to be fixed, and our results suggest that their value is not very important. For instance, the article notes that the value of α rarely comes into play:

> *We avoid the possibility of* nearly canceling roots *in parameter initializations by requiring the minimum Euclidean distance between inverted polynomial roots to be greater than α. This is done in line 4 of Algorithm 1, though the condition is rarely triggered if the order of the model is of typical size ($p, q < 4$).*

> *17. Does the improved inference primarily help in estimation, or does it consistently translate into better predictive performance as well? Were forecasts generated using their algorithm compared with those from classical ARMA implementations (e.g., Box-Jenkins, Kalman filter-based methods)?*

The primary goal is improved estimation. While this has the potential to lead to better predictive performance, we have found that predictive performance is relatively unchanged with the improved estimation. This is primarily a result of the fact that all ARMA model predictions correspond to a Gaussian distribution, and the mean and variance of future points are fairly stable, even under

different selections of model sizes. The larger practical impact is highlighted in the discussion and the last paragraph of section 3.1, which is that improved likelihood can result in better confidence intervals, hypothesis tests, and inference:

> *Among the most common motivations for fitting ARMA models is to model serial correlations in a regression model; in this setting, the discovered shortcomings in log-likelihood are often enough to change the outcome of the analysis... subtracting the reported log-likelihood deficiencies (which has a median value of* 0.66*) of existing software to either or both* ll_0, ll_1 *could change the outcome of this test.*

The Box-Jenkins approach and Kalman Filters play the exact same role in our approach as existing implementations. These remain unchanged, we just use combine these ideas / techniques while also considering multiple parameter initializations.

> *18. Were the improvements in forecasting found to be statistically significant? Was any test (e.g., Diebold-Mariano) used to validate forecast accuracy?*

As noted in the response to question 17, impacts to forecasting accuracy is not a focus of our manuscript.

> *19. Were forecasting accuracy metrics—such as RMSE, MAE, MAPE, or out-of-sample log-likelihood—used in the evaluation? What forecasting horizons (e.g., 1-step, multi-step) were considered when comparing the improved MLE ARMA with the traditional approach?*

As noted in the response to question 17, impacts to forecasting accuracy is not a focus of our manuscript.

References

1. Chen WW, Deo RS. The restricted likelihood ratio test for autoregressive processes. Journal of Time Series Analysis. 2012;33(2):325–339.

2. Monahan JF. Fully Bayesian analysis of ARMA time series models. Journal of Econometrics. 1983;21(3):307–331.

3. Chib S, Greenberg E. Bayes Inference in Regression Models with ARMA (p, q) Errors. Journal of Econometrics. 1994;64(1):183–206.

4. Chon KH, Cohen RJ. Linear and Nonlinear ARMA Model Parameter Estimation using an Artificial Neural Network. IEEE Transactions on Biomedical Engineering. 1997;44(3):168–174.

5. Chen WW, Deo RS. Bias reduction and likelihood-based almost exactly sized hypothesis testing in predictive regressions using the restricted likelihood. Econometric Theory. 2009;25(5):1143–1179.

6. Chen WW, Deo RS. Weighted least squares approximate restricted likelihood estimation for vector autoregressive processes. Biometrika. 2010;97(1):231–237.

7. Weiß CH, Zhu F. Mean-Preserving Rounding Integer-Valued ARMA Models. Journal of Time Series Analysis. 2024;46:530–551.

8. Chakhchoukh Y. A New Robust Estimation Method for ARMA Models. IEEE Transactions on Signal Processing. 2010;58(7):3512–3522.

9. Lii KS. Identification and Estimation of non-Gaussian ARMA Processes. IEEE Transactions on Acoustics, Speech, and Signal Processing. 1990;38(7):1266–1276.

10. Le Cam L. Maximum Likelihood: An Introduction. International Statistical Review / Revue Internationale de Statistique. 1990;58(2):153–171.

11. Pankratz A. Forecasting with Univariate Box-Jenkins Models. In: Wiley Series in Probability and Statistics. Wiley; 2008. p. 576.

12. Shumway RH, Stoffer DS. Time Series Analysis and Its Applications: With R Examples. Springer Texts in Statistics. Cham: Springer International Publishing; 2017.
