# Supplementary material for: Revisiting inference for ARMA models: Improved fits and superior confidence intervals
Source: PLoS One. 2025 Oct 24;20(10):e0333993. doi: 10.1371/journal.pone.0333993 (PMC12551883; doi:10.1371/journal.pone.0333993)
Supplement: Attachment — Submitted filename: response-to-reviewers_auresp_2.pdf [file pone.0333993.s007.pdf]

Reviewer 1

> *This research is written in standard english, Also this research is technically sound and all the statistical analysis is done properly.*

Thank you for your review of our manuscript. We would like to highlight to the editor that this comment from the reviewer does not match what the reviewer answered for the question: “Is the manuscript technically sound, and do the data support the conclusions?”. Given this comment, we suspect the reviewer’s response “no” to this question was done accidentally, and kindly ask the editor to consider this reviewers comment as evidence that the reviewer does agree that the manuscript is technically sound.

Referee 2

> *The paper has been adequately revised, in my opinion and is suitable for publication. The authors should consider a deeper investigation of REML in their future work.*

Thank you once again for your thoughtful review of our manuscript. We will indeed conduct a deeper investigation of REML in the future, and will consider adding this to our software package.

Referee 4

This paper addresses a subtle but widespread flaw in statistical practice. The proposed solution is innovative, reproducible, and impactful. With small refinements, it can become a go-to reference for robust ARMA inference. the paper can be accepted with minor revision.

Thank you for the review of our paper, we appreciate the time and thought you put into the article to help us make it stronger. In this current review, you recommend a few minor refinements before publication. After some serious thought, we do not feel that the recommended changes are improvements. Instead, we ask the reviewer and editor to consider the article without making these additional changes. Below, we give detailed and itemized responses to each of the reviewers comments and suggestions.

1. While processing time is discussed, more formal benchmarking across software environments (e.g., R vs Python) would help practitioners decide when to use these methods.

As a reminder to the reviewer, though our algorithm is language agnostic, it has thus far only been implemented in R. The request for formal benchmarking across software environments (e.g., R vs Python) is therefore not relevant to the current work. Specifically, it is not immediately possible to compare computing times of the implemented algorithm in R versus Python, as the Python implementation does not yet exist. Implementing the algorithm in any alternative programming language (such as Python) is not a reasonable request nor is it within the scope of this paper.

Though software computing times is not the focus of our work and is only tangentially related to our manuscript, we still provide an extensive summary of the performance of our implemented algorithm in R relative to the standard alternative. Our previous round of revisions resulted in Table 1, which summarizes the computational time for various combinations of data size and algorithm choice. This table is a result of summarizing millions of simulations, and any additional information beyond what we already provide will be a distraction from the main argument of the manuscript.

2.The focus is primarily on Gaussian noise. The authors mention non-Gaussian models in passing—some brief results or commentary on how the proposed methods extend to robust or non-Gaussian ARMA models would enhance generalizability.

We focus on Gaussian noise because this is the current standard practice that enables likelihood based inference for ARIMA models. Algorithms for maximum likelihood estimation of ARIMA model parameters with more general noise are not readily available or used in practice. Whether or not a practitioner is aware of this, the assumption of Gaussian errors is made implicitly by all general purpose software—that we are aware of—for fitting ARIMA models. Though it is considered a niche topic, we do include sufficient references for readers to find more information about possible alternatives to this assumption, such as [1]. This point is emphasized in the current manuscript.

... we focus on the approach most widely adopted in statistical practice: likelihood maximization, under the assumption that the WN process $\{w_t\}$ is Gaussian.

The assumption of Gaussian errors is not strictly necessary for randomly initializing model parameters. In this regard, the algorithms we propose can be generalized to non-Gaussian models, even though they are not used in practice. The Gaussian assumption is, however, required for parameter estimation. This is because the numeric optimization routine that is used to fit ARMA

model parameters [2] relies on the Kalman filter, which is a method that can be used to evaluate the likelihood of a linear Gaussian state space model. The Kalman filter, and its use in fitting ARIMA model parameters, is a topic that is widely covered elsewhere, including several references that we already make.

In summary, our initialization schemes (Lines 3–15 of Algorithm 1 and Lines 3–19 of Algorithm 2) describe algorithms for randomly sampling causal and invertible ARMA model parameters, which has no relation to the assumption of Gaussian errors in the model. However, the currently accepted standard practice of parameter estimation for ARMA models involves the use of a Kalman filter, which assumes that the errors are Gaussian. Other probability models for the errors are not generally used because off-the-shelf algorithms for maximum likelihood estimators for general models are not widely available. Because non-Gaussian ARMA models are a niche topic and not widely employed in practice, and because we already provided references to relevant papers on the topic, we do not believe that any additional discussion on the topic is interesting or helpful to readers.

> *3. The choice of stopping criterion (no new maximum in M steps) is somewhat heuristic. A more adaptive or data-driven rule, or guidance on setting M based on sample size or model order, would be helpful.*

It's important to highlight that our proposed algorithm allows for arbitrary stopping criteria, as it is an input (rather than requirement) of our function. If users have other preferred stopping criteria, then that still falls within our recommendations provided in this article, and we already provide suitable references for users to find alternatives if they wish to do so.

The default stopping criteria that we highlight and recommend is indeed somewhat heuristic, but this is a fact that we already note in the article.

> *Alternative stopping criterion can be used (see for example, [3]), but we found that this simple heuristic works well in practice.*

One reason that this stopping criteria is so effective is because the default initialization typically results in the algorithm converging to the global maximum. In some cases, this default initialization does result in local maximums, in which case we find only one or two initializations sufficient to fix the issue.

The recommended stopping criteria is also both "adaptive" and "data-driven", as model / data combinations that have more local maximums will continue restarting the count in the stopping criteria until new parameter estimates do not have higher likelihood than those previously found.

Other approaches that are less heuristic, such as those highlighted in [3], often require many hundreds or thousands of initializations in order to stop, even in the scenario where all initializations lead to the same parameter estimate. Though these types of estimates have support in some situations, we find that they lead to a large amount of unecessary computation for ARIMA models.

Additional guidance for setting M based on sample size or model order is provided already in Figure 4 of the article. In this figure, we see that even in the most difficult scenarios (large amounts of parameter redundancy), the default choice of $M = 10$ generally is sufficient to fix the consistency of most AIC tables.

4.Figures mentioned (e.g., Figs 1–8) are informative, but it would be useful to annotate likelihood surfaces more explicitly in plots to show how initializations evolve toward the global maximum.

Thank you for your suggestion. We appreciate the value of visualizing likelihood surfaces and optimizer trajectories. However, even the most basic examples considered in this work (e.g., AR(1) or MA(1) models) have a three-dimensional likelihood surface or higher. This dimensionality makes direct and informative visualization infeasible for our cases of interest. Additionally, our optimization routines are implemented in C for computational efficiency, and we do not retain intermediate states during optimization. Capturing and plotting the evolution of initializations toward the global maximum would require significant changes to our implementation, likely with substantial impacts on runtime and memory usage.

Finally, illustrations of the behavior of standard optimization algorithms (such as BFGS) are readily available elsewhere. The annotation of how the initializations evolve towards the global maximum would indeed be interesting, but is not readily doable or necessary for our purpose. As our article makes clear, we use established numerical methods rather than proposing new optimization techniques. Including such figures would therefore not provide novel insights in the context of our contribution.

5. Though the method improves likelihoods, a brief reflection on whether overfitting could result (especially in small samples with complex models) would strengthen the discussion.

You raised the question of the potential for over-fitting in the previous review. As such, we would like to address the question as clearly as possible, at the expense of being verbose.

First and foremost, our algorithm does not increase model complexity compared to existing alternatives. Our method is simply an alternative approach to parameter estimation for the same fixed model; no additional parameters or sources of flexibility are introduced, as the specified model structure remains unchanged. Overfitting is a result of using an overly complex model (e.g., too many model parameters) to a given dataset, so that the model learns features of the particular sample and cannot generalize well to new samples. Because our algorithm does not add any additional model complexity compared to the standard approach, it cannot result in any additional overfitting.

What distinguishes our algorithm from the standard approach is the ability to better optimize the model likelihood, correcting the cases where existing algorithms are empirically shown to fail at this task. Improving optimization within the same model cannot itself lead to overfitting compared to existing estimators; it simply provides better parameter estimates for the specified model. For instance, consider fitting an ARMA$(2, 1)$ model to a fixed dataset. Regardless of the estimation procedure, we must estimate five parameters (AR and MA parameters, intercept, and a variance parameter). Our method fits these same parameters—no more or fewer—while providing improved likelihood optimization. Thus, the resulting model is not more complex or prone to overfitting than other alternatives.

Overfitting may occur for, for instance, if values of p and q are chosen to be too large for a particular data set. However, for ARMA models, overfitting is generally less concerning than other issues that arise with large values of p and q, such as canceling roots and identifiability issues that arise and are discussed in our manuscript. Additionally, selecting model size is not a topic that we discuss in our

article as it is well documented elsewhere [4, 5, 6, 7]. In addition to a demonstration and description of using AIC for model selection, we include the following paragraph that describes techniques for model selection, which can be used to avoid selecting models with unnecessarily large values of p and q.

> *Minimizing the AIC (or an alternative information criterion) is not the only accepted approach to order selection. A classical perspective on model selection involves consulting sample autocorrelation plots, partial autocorrelation plots, conducting tests such as Ljung-Box over various lags, studying the polynomial roots of fitted models, and checking properties of the residuals of the fitted models [4, 7, 5]. This approach helps avoid fitting models that are possibly over-parameterized. However, additional computational power and increasing volumes of data have favored automated data analysis strategies that fit many models and evaluate them using a model selection criterion. In principle, a simple model selection criterion such as AIC can address parsimony and guard against over-parameterization as well. Diagnostic inspection can be combined with these automated approaches. For example, a table of AIC values can be generated, and models with promising likelihoods can be explored further [7].*

In summary, overfitting is a topic that is not directly related to our manuscript. Despite this, the current manuscript provides several examples and references on model selection that address this concern, and as a result, additional discussion on the topic is not warranted or beneficial.

> *6. The manuscript is long but well-organized. Still, trimming some of the implementation details from the main text to Supplementary Material could help readability.*

We disagree with the assertion that this article is long, particularly for the given topic and venue. PLOS ONE does not have any formal requirements on page length. Even including 8 large figures and three tables, the article is only 17 pages long, which is well below the average that we have observed at PLOS ONE. Our original submission did have fewer implementation details, but reviewers from our previous round of revisions requested that we add more details, and we therefore complied. As paper length is not a formal requirement, and because you state that the paper is well-organized, we feel there is no need to modify the length of the paper in order to improve readability.

> *7. Cite and contrast with more recent Bayesian optimization or machine learning-based approaches to global optimization for time series models.*

The suggestion to contract with Bayesian methodologies was provided in your last review, and we feel that we adequately responded to this concern in our most recent submission. To reiterate these points for clarity, a direct comparison to Bayesian algorithms for ARMA estimation is not relevant to our current study. Our goal is to highlight and address an unknown shortcoming in maximum likelihood estimation for a commonly used model; Bayesian optimization is not related at all to this task, and as such there is no way to meaningfully compare to our approach as requested. For readers that are interested in fitting an ARIMA model using Bayesian techniques, we have already included the most relevant citations [8, 9].

It is not clear what is meant by "global optimization for time series models", or how one could meaningfully compare our work to other machine-learning based approaches for time series. There are incredibly diverse models that can be used for time series analysis, not all of which have the

same purpose. You may be requesting that we compare the predictive ability of ARIMA models to other types of models; this type of comparison has been done elsewhere, and is not relevant to our goal of fixing optimization issues for fitting ARIMA models.

References

1. Lii KS. Identification and Estimation of non-Gaussian ARMA Processes. IEEE Transactions on Acoustics, Speech, and Signal Processing. 1990;38(7):1266–1276.

2. Gardner G, Harvey AC, Phillips GDA. Algorithm AS 154: An Algorithm for Exact Maximum Likelihood Estimation of Autoregressive-Moving Average Models by Means of Kalman Filtering. Journal of the Royal Statistical Society Series C (Applied Statistics). 1980;29(3):311–322.

3. Hart WE. Sequential Stopping Rules for Random Optimization Methods with Applications to Multistart Local Search. SIAM Journal on Optimization. 1998;9(1):270–290.

4. Box G, Jenkins G. Time Series Analysis: Forecasting and Control. San Francisco, Holdan-Day; 1970.

5. Shumway RH, Stoffer DS. Time Series Analysis and Its Applications: With R Examples. Springer Texts in Statistics. Cham: Springer International Publishing; 2017.

6. Akaike H. A New Look at the Statistical Model Identification. IEEE Transactions on Automatic Control. 1974;19(6):716–723.

7. Brockwell PJ, Davis RA. Time Series: Theory and Methods. Springer Series in Statistics. New York, NY: Springer; 1991.

8. Monahan JF. Fully Bayesian analysis of ARMA time series models. Journal of Econometrics. 1983;21(3):307–331.

9. Chib S, Greenberg E. Bayes Inference in Regression Models with ARMA (p, q) Errors. Journal of Econometrics. 1994;64(1):183–206.
